# Selective function of the PDZ domain of Dishevelled in noncanonical Wnt signalling

**DOI:** 10.1242/jcs.259547

**Published:** 2022-05-31

**Authors:** Juliusz Mieszczanek, Helen Strutt, Trevor J. Rutherford, David Strutt, Mariann Bienz, Melissa V. Gammons

**Affiliations:** 1 MRC Laboratory of Molecular Biology, Cambridge Biomedical Campus, Francis Crick Avenue, Cambridge, CB2 0QH, UK; 2 University of Sheffield, School of Biosciences, Firth Court, Western Bank, Sheffield, S10 2TN, UK

**Keywords:** Dishevelled, PDZ domain, Noncanonical Wnt signalling, Planar cell polarity, *Drosophila*

## Abstract

Dishevelled is a cytoplasmic hub that transduces Wnt signals to cytoplasmic effectors, which can be broadly characterised as canonical (β-catenin dependent) and noncanonical, to specify cell fates and behaviours during development. To transduce canonical Wnt signals, Dishevelled binds to the intracellular face of Frizzled through its DEP domain and polymerises through its DIX domain to assemble dynamic signalosomes. Dishevelled also contains a PDZ domain, whose function remains controversial. Here, we use genome editing to delete the PDZ domain-encoding region from *Drosophila dishevelled*. Canonical Wingless signalling is entirely normal in these deletion mutants; however, they show defects in multiple contexts controlled by noncanonical Wnt signalling, such as planar polarity. We use nuclear magnetic resonance spectroscopy to identify bona fide PDZ-binding motifs at the C termini of different polarity proteins. Although deletions of these motifs proved aphenotypic in adults, we detected changes in the proximodistal distribution of the polarity protein Flamingo (also known as Starry night) in pupal wings that suggest a modulatory role of these motifs in polarity signalling. We also provide new genetic evidence that planar polarity relies on the DEP-dependent recruitment of Dishevelled to the plasma membrane by Frizzled.

## Abstract (precis)

**Summary:** The PDZ domain of *Drosophila* Dishevelled functions in noncanonical Wnt signalling but is dispensable for signalling through Armadillo, the fly homologue of β-catenin.

## INTRODUCTION

Wnt signalling pathways are conserved ancient cell communication networks that regulate cell fate determination and differentiation during development (Clevers, 2006; Gammons and Bienz, 2018; Logan and Nusse, 2004). All Wnt signals pass through the cytoplasmic hub protein Dishevelled, where signals diverge into distinct branches to specify various outputs (Wallingford and Habas, 2005). These are broadly defined as canonical (β-catenin dependent), typically promoting cellular proliferation or differentiation (Bienz and Clevers, 2003; MacDonald et al., 2009), and noncanonical, a collection of less well-defined signalling branches. One of these is the planar cell polarity (PCP) pathway, which coordinates cell orientation within epithelia and morphogenetic processes such as convergent extension (Wallingford et al., 2002) (Theisen et al., 1994) (Shulman et al., 1998). Dishevelled contains three conserved domains called DIX (Dishevelled and Axin), PDZ (post-synaptic density protein-95, Disc large tumor suppressor, zonula occludens-1) and DEP (Dishevelled, EGL-10 and pleckstrin). The DEP domain binds to the intracellular face of Frizzled seven-pass transmembrane receptors (Gammons et al., 2016a; Tauriello et al., 2012), while the DIX domain undergoes head-to-tail polymerisation to assemble dynamic signalosomes (Schwarz-Romond et al., 2007), but the function of the PDZ domain remains controversial.

PDZ domains are small globular protein–protein interaction modules (Ponting, 1997) that recognise PDZ-binding motifs (PBMs) typically at the C terminus of their binding partners (Saras and Heldin, 1996) through a peptide-binding groove (Lee and Zheng, 2010). Initially, evidence suggested that the Dishevelled PDZ domain binds directly to a highly conserved KTxxxW motif (where x indicates any amino acid) in the cytoplasmic portion of Frizzled (Wong et al., 2003) that is essential for Wnt signal transduction to β-catenin (Umbhauer et al., 2000) and noncanonical responses (Wu et al., 2008). However, it is now accepted that the DEP rather than the PDZ domain is the ligand of Frizzled: for example, the minimal DEP domain is recruited to the plasma membrane by Frizzled paralogues upon co-overexpression in cells (Gammons et al., 2016a; Pan et al., 2004; Tauriello et al., 2012), and *dsh^1^* mutant flies, which bear a point mutation in the Frizzled-binding loop of the DEP domain, show reduced recruitment of Dishevelled to the apicolateral membrane (Axelrod, 2001; Shimada et al., 2001). Furthermore, the recently obtained crystal structure of human frizzled-4 (FZD4) has revealed that the KTxxxW motif is contained within a structured α-helix that extends underneath the membrane (Yang et al., 2018) and is presumably anchored to it, as occurs in the related G-protein-coupled membrane receptors (Venkatakrishnan et al., 2013), which is incompatible with PDZ binding as demonstrated by nuclear magnetic resonance (NMR) (Gammons et al., 2016b).

There have been numerous attempts to pin down the function of the Dishevelled PDZ domain in the transduction of Wnt signals, without reaching a clear consensus. Some have argued that this domain is required for both canonical and noncanonical signalling (Sokol, 1996; Tada and Smith, 2000; Yanagawa et al., 1995), whereas others have concluded that its function is limited to different subsets of noncanonical signalling responses (Axelrod et al., 1998; Rothbacher et al., 2000; Umbhauer et al., 2000; Habas et al., 2003). The likely reason for these discrepancies is that these studies were based on overexpression of Dishevelled, which activates Wnt-independent signalling through β-catenin but also interferes with PCP and other noncanonical signalling responses in vertebrate and invertebrate tissues (Boutros et al., 1998; Park et al., 2005; Penton et al., 2002). Therefore, a definitive conclusion can only be obtained from analyses that are based on PDZ deletions of endogenous genes encoding Dishevelled; however, this is highly challenging in vertebrates since their genomes encode three Dishelleved paralogues (DVL1–DVL3). Therefore, we turned to *Drosophila*, which has a single *dishevelled* (*dsh*) gene, to investigate the function of the Dishevelled PDZ domain in multiple developmental contexts following its deletion using CRISPR/Cas9.

Here, we show that the PDZ domain of *Drosophila* Dsh is dispensable for canonical Wingless (Wg) signalling but is essential in multiple cellular contexts controlled by noncanonical signalling. Focussing on PCP in the pupal wing, we found that the PDZ domain is required for the asymmetric accumulation of Dsh itself, and of Flamingo (Fmi; also called Starry night, Stan), a cadherin-like transmembrane protein required for PCP signalling (Chae et al., 1999; Usui et al., 1999). Furthermore, we used NMR spectroscopy to identify PBMs at the C termini of several components of the distal PCP complex that bind specifically to the binding cleft of the Dishevelled PDZ domain. Truncations of these cognate PBMs by CRISPR/Cas9 produce changes in the proximodistal distribution of Flamingo, which suggests a modulatory role of these PBMs in PCP signalling. We also report a new *fz* allele that illustrates the importance of the DEP-dependent interaction between Frizzled and Dishevelled in planar cell polarity.

## RESULTS AND DISCUSSION

### Deletion of the Dsh PDZ domain causes PCP defects

**Fig. 1.**
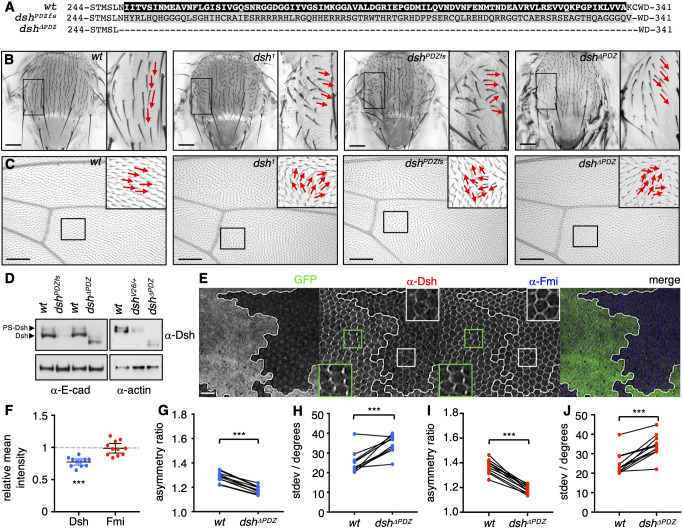
***Dsh^ΔPDZ^* flies show PCP defects.** (A) Amino acid sequence alignment of wt Dsh and the *dsh^PDZfs^* and *dsh^ΔPDZ^* mutants. The wt PDZ domain is indicated by the black box, and the frame-shifted sequence in *dsh^PDZfs^* is shown in grey. (B,C) Images of adult flies showing orientation of thoracic bristles (B, red arrows) or wing hairs (C, red arrows). Boxes indicate regions shown in inset images. Scale bars: 150 μm. Images are representative of 12 flies. (D) Western blot analysis of extracts from late third-instar larval imaginal discs (left) or 28 h APF pupal wings (right), showing levels of Dsh and PS-Dsh. E-cadherin and actin were used as loading controls. Blots are representative of more than three experiments. (E) Pupal wings carrying *dsh^ΔPDZ^* mutant clones marked by loss of GFP, immunolabelled for Dsh (red) and Fmi (blue). The boxes mark regions shown in magnified views (insets). Clone boundaries are marked with white lines. Scale bar: 10 μm. (F–J) Quantitation of (F) relative mean intensities of anti-Dsh and anti-Fmi antibody staining in *dsh^ΔPDZ^* mutant tissue compared to that in wt tissue, and (G,I) mean polarity and (H,J) variation in polarity angle for (G,H) Dsh and (I,J) Fmi (*n*=11 wings). Error bars in F are 95% confidence intervals; two-tailed paired *t*-tests were used to compare values in the same wing, ***P<0.001.

To delete the PDZ-encoding sequences from *Drosophila dsh* using CRISPR/Cas9 gene engineering, we designed two guide RNAs (gRNAs) targeting either side of the PDZ domain to generate a frame-shift deletion (*dsh^PDZfs^*) or a clean excision (*dsh^ΔPDZ^*) (Fig. 1A). Both mutants were homozygous viable and failed to exhibit canonical Wg defects in the larval cuticle (Nusslein-Volhard and Wieschaus, 1980). However, both mutants were defective for PCP, showing misorientation of bristles on the dorsal thorax and of wing hairs in adult flies, like *dsh^1^* mutants (Theisen et al., 1994) (Fig. 1B,C).

*dsh^1^* mutants also show reduced levels of phosphorylated Dsh (which is detectable as a series of upward-shifted bands by gel electrophoresis; referred to here as PS-Dsh) and reduced association of Dsh with Frizzled at the apicolateral plasma membrane (Axelrod, 2001). Therefore, we assessed PS-Dsh in our new mutants by western blotting of protein extracts from third-instar larval imaginal discs or pupal wings. For both *dsh^ΔPDZ^* and *dsh^PDZfs^* mutants, as in wild-type (wt) controls, PS-Dsh was clearly visible (Fig. 1D). However, the total levels of Dsh were reduced more than 10-fold in *dsh^PDZfs^* mutants. In contrast, total Dsh levels were only mildly reduced in *dsh^ΔPDZ^* mutants (∼2-fold), similar to those in *dsh^V26^* heterozygotes (*dsh^V26/+^*; Fig. 1D), which do not show PCP phenotypes (Klingensmith et al., 1994). Therefore, it is unlikely that these mildly reduced Dsh levels are responsible for the PCP defects in the *dsh^ΔPDZ^* mutants.

By immunolabelling early pupal wings with an anti-Dsh antibody, we confirmed that Dsh accumulates at proximodistal cell edges in wt tissue, as reported previously (Axelrod, 2001) (Fig. 1E). In *dsh^ΔPDZ^* mutant clones, anti-Dsh staining remained pronounced along the cell membranes (Fig. 1E), consistent with the presence of PS-Dsh in the mutants, although the staining intensity was reduced compared to that in neighbouring wt cells (Fig. 1F), as expected from the lower levels of Dsh in the *dsh^ΔPDZ^* mutant (Fig. 1D). Importantly, the Dsh signals were no longer asymmetrical within cells (Fig. 1G,H). The same was true for Fmi, whose asymmetrical accumulation was observed in wt clones but not in *dsh^ΔPDZ^* mutant clones (Fig. 1I,J), even though the intensity of anti-Fmi staining remained unchanged in the latter (Fig. 1F). This implicates the PDZ domain of Dsh in the asymmetrical accumulation of these two proteins at proximodistal cell edges and suggests that this domain functions in the assembly of distal polarity complexes (Strutt et al., 2016).

### *dsh^ΔPDZ^* mutant flies show defects in axon guidance and leg morphogenesis

**Fig. 2.**
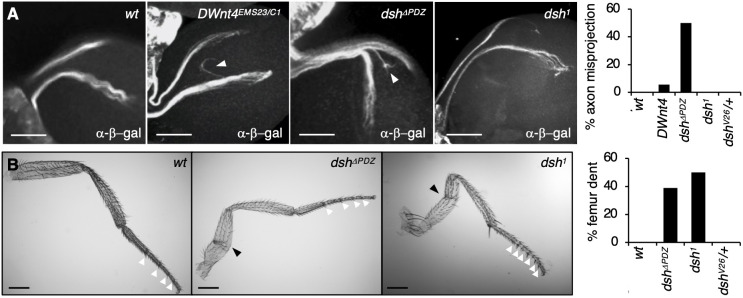
***Dsh^ΔPDZ^* flies show retinal axon and leg defects.** (A) Dorsal- and ventral-most retinal axons visualised in the indicated strains with *omb-lacZ* using anti-β-galactosidase immunolabelling. Misrouted axons are indicated by white arrowheads, and quantification of percentage axon misrouting is shown on the right. *n*>10. Scale bars: 50 μm. (B) Images of wt and malformed legs, characterised by femur dent (black arrowheads). The percentage of dented femurs in the indicated strains is shown on the right (*n*>10). Complete and partial joints between tarsal segments are indicated by white arrowheads. Scale bars: 200 μm.

Next, we assessed *dsh^ΔPDZ^* flies for defects in other Dsh-dependent noncanonical processes, including dorsal closure, head involution (Kaltschmidt et al., 2002) and border cell migration during oogenesis (Bastock and Strutt, 2007), but no defects were observed in these processes (J.M., unpublished). We then examined retinal axon guidance, which depends on Wnt4 and Fz2 but not on Armadillo, the *Drosophila* homologue of β-catenin (Sato et al., 2006). Using *omb-lacZ* as a marker for dorsal and ventral retinal axons in larvae (Sato et al., 2006), we found that *dsh^ΔPDZ^* mutants frequently showed misrouting of axons (in 5/10 larvae). This is in contrast to wt dorsal and ventral axons, which invariably projected (20/20 larvae) to the dorsal and ventral lamina, respectively (Fig. 2A), as in *dsh^V26/+^* larvae (20/20 larvae), confirming that the defect in the *dsh^ΔPDZ^* larvae is not due to reduced Dsh protein levels. *dsh^1^* larvae also showed normal axons (10/10 larvae) (Fig. 2A), suggesting that retinal axon guidance depends on a noncanonical signalling branch distinct from PCP. Indeed, retinal axon guidance involves Wnt4 and Fz2 (Sato et al., 2006), whereas PCP does not require Wnts (Ewen-Campen et al., 2020; Yu et al., 2020) and acts through Fz rather than Fz2 (Boutros et al., 2000; Strapps and Tomlinson, 2001).

We also observed malformed leg femurs in many *dsh^ΔPDZ^* homozygous flies (7/18 flies), as also seen in *dsh^1^* flies (7/14 flies) (Fig. 2B). However, the extra tarsal joints previously reported for mutants in the planar polarity gene *fz* (Held et al., 1986; Strutt et al., 2012) were seen only in *dsh^1^* but not in *dsh^ΔPDZ^* flies (Fig. 2B). Leg morphogenesis depends on RhoA (also known as Rho1) (Halsell et al., 2000), and since *RhoA* genetically interacts with *dsh* and *fz* (Strutt et al., 1997), we tested *dsh^ΔPDZ^* and *dsh^1^* for a genetic interaction with the *RhoA*-null allele *RhoA^72O^*: indeed, double heterozygosity for *dsh^ΔPDZ^* or *dsh^1^* and *RhoA^72O^* increased the penetrance of abnormal femurs from 1% to 7% or 19%, respectively.

Thus, our loss-of-function studies of *dsh^ΔPDZ^* mutants have uncovered a role for the Dishevelled PDZ in three developmental contexts that require noncanonical Wnt signalling, namely PCP, retinal axon guidance and leg morphogenesis. Furthermore, our results demonstrate conclusively that the PDZ domain is dispensable for canonical Wg signalling, even when Dsh protein expression levels are significantly reduced. This is consistent with previous findings from human cell-based complementation assays based on re-expression of DVL2 at endogenous levels (Gammons et al., 2016b) and from rescue assays in *Drosophila* based on complementation by overexpressed Dsh deletion constructs (Axelrod et al., 1998).

### The Dishevelled PDZ domain binds to multiple PCP components

Several PCP components contain putative PBMs in their C termini (i.e. short conserved motifs that bind to the PDZ groove; Songyang et al., 1997), namely Fmi (Strutt et al., 2019), Fz, Dgo and Dsh itself (Lee et al., 2015). We therefore tested whether any of these proteins bind to the Dishevelled PDZ domain using a sensitive *in vitro* binding assay based on NMR spectroscopy (Gammons et al., 2016b). As attempts to purify the PDZ of fly Dishevelled (Dsh_PDZ_) were unsuccessful, we chose the PDZ domain of human DVL2 (DVL2_PDZ_), which shares 91.8% sequence similarity with Dsh_PDZ_, including complete conservation of the ligand-binding cleft residues (Fig. S1). After bacterial expression and purification, we acquired ^1^H–^15^N correlation spectra of 100 µM ^15^N-labelled DVL2_PDZ_ alone or after incubation with 300 µM individual purified PBMs tagged with lipoyl (Lip).

**Fig. 3.**
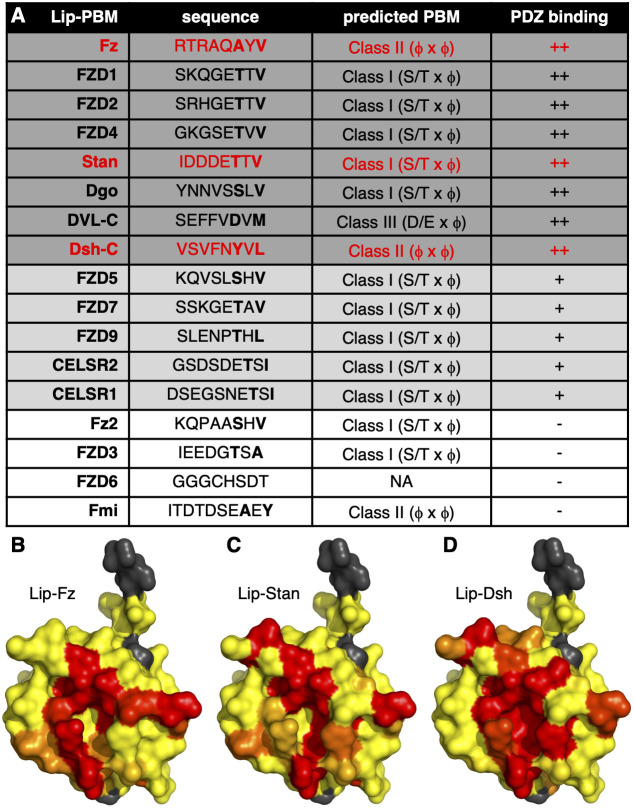
**PDZ-binding peptides in distal polarity proteins.** (A) Table summarising DVL2_PDZ_ binding for lipoyl-tagged PBMs (Lip–PBM) from the indicated proteins: ++, strong; +, modest; −, none. PDZ-binding motif residues are shown in bold. Selected strong binders are highlighted in red. X, any amino acid; φ, hydrophobic amino acid; NA, not assigned. (B–D) Heat maps of line broadening and chemical shift perturbation upon binding of (B) Lip–Fz, (C) Lip–Stan and (D) Lip–Dsh (yellow, less than mean; red, greater than mean; threshold, 1× s.d.), projected on to the crystal structure of DVL2_PDZ_ (PDB:2REY; grey, prolines and unassigned peaks).

We found that Lip–Fz but not Lip–Fz2 caused a small number of chemical shift perturbations (CSPs) in PDZ backbone N–H resonances (Fig. S2), indicating that Fz, but not Fz2, contains a functional PBM. Fz is the predominant Frizzled protein implicated in *Drosophila* PCP signalling (Boutros et al., 2000; Strapps and Tomlinson, 2001). Of the Lip-tagged human Frizzled paralogues, FZD4, FZD1 and FZD2 bound strongly; FZD5, FZD7, FZD9 and FZD10 bound moderately; whereas FZD3 and FZD6 showed no binding (Fig. 3A) even though FZD3 contains a clear match to a class I PBM. We next generated a ‘heat map’, projecting Lip–Fz CSPs onto the crystal structure of DVL2_PDZ_ (PDB:2REY), whose resonances have been assigned previously (Gammons et al., 2016b), to confirm that the residues affected by PBM interactions are located predominantly in the αB/βB peptide-binding groove (Fig. 3B).

Fmi has two isoforms with alternative C termini generated by alternative splicing (Chae et al., 1999; Usui et al., 1999), one of which has a predicted class II PBM at its C terminus, but this motif failed to bind (Fig. 3A). However, the other isoform (referred to hereafter as Stan) with a predicted class I C-terminal PBM (Strutt and Strutt, 2008) was positive for PDZ binding in our NMR assay. Moreover, the human Fmi and Stan orthologues CELSR1 and CELSR2 both bound moderately (Fig. 3A). Chemical shift mapping confirmed that the Stan PBM also binds to the classical PBM-binding groove (Fig. 3C).

Another distal PCP protein with a putative PBM is Dgo, which was also positive for PDZ binding in our NMR assay (Fig. 3A). However, this PBM is not conserved in the human orthologue diversin (also known as ANKRD6), whose binding to Dishevelled has been mapped to the DEP domain (Moeller et al., 2006; Schwarz-Romond et al., 2002). Finally, we also confirmed that the C-terminal PBM of Dsh binds to the DVL2 PDZ domain (Fig. 3A,D), as has been reported previously for DVL1 (Lee et al., 2015).

These NMR assays demonstrate that the Dishevelled PDZ domain can bind to multiple ligands, which cannot be predicted reliably by their sequence alone (Fig. 3A). Remarkably, our systematic NMR screen revealed cognate PBMs in all four components of the distal planar polarity complex (Fz, Stan, Dgo and Dsh) and therefore identified each of these as candidate physiologically relevant ligands of Dishevelled PDZ.

### PBM truncations of PCP genes modulate the polar distribution of Fmi

**Fig. 4.**
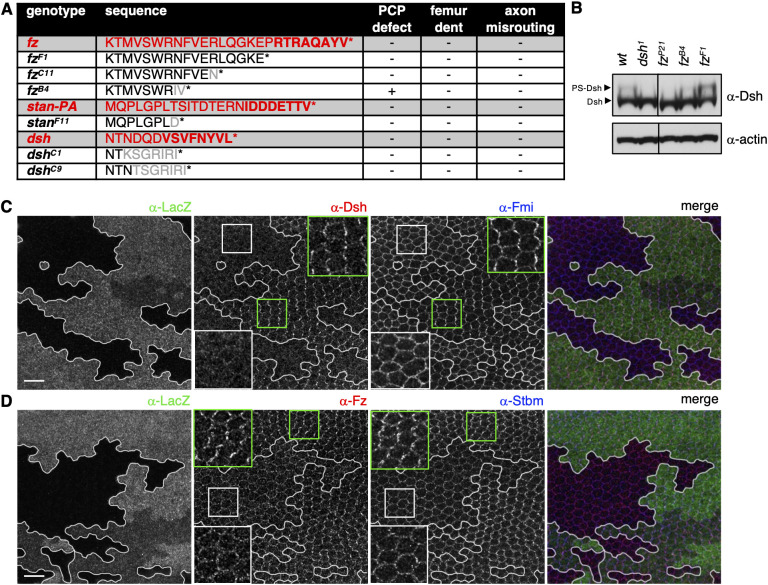
**PBM truncations of distal polarity genes.** (A) Table summarising wt (red) or mutant alleles (black) with their C-terminal sequences (bold, PDZ-binding motif; grey, mutated residues) and associated phenotypes (+, mutant phenotype; −, wild type). (B) Western blot analysis of Dsh and PS-Dsh in imaginal disc extracts from the indicated strains. Actin was used as a loading control. Blots are representative of three experiments. (C,D) Pupal wings carrying *fz^B4^* clones of cells marked by loss of β-galactosidase (LacZ) and immunolabelled for (C) Dsh and Fmi or (D) Fz and Stbm, as indicated. Red and blue colours, respectively, in merge images. The boxes mark regions shown in magnified views (insets). Clone boundaries are marked with white lines. Images are representative of 12 experiments. Scale bars: 10 μm.

To test the functional relevance of these PBMs during *Drosophila* development, we truncated Fz, Stan and Dsh upstream of their PBMs using CRISPR/Cas9. We did not target the Dgo PBM because homozygous null *dgo^380^* flies failed to phenocopy the bristle misorientation defects in the dorsal thorax (Feiguin et al., 2001) (J.M., unpublished). For Stan, we generated a single allele by introducing a point mutation (T3563D) followed by a stop codon (*stan^F11^*). *stan^F11^* flies were homozygous viable and failed to show any of the above-described mutant phenotypes (Fig. 4A). For Dsh, we isolated two alleles with a frame shift after T611 (*dsh^C1^*) or N612 (*dsh^C9^*), both of which result in a unique class II PBM that is, however, unable to bind DVL2 PDZ (Fig. S2). Both of these mutants were viable and aphenotypic (Fig. 4A). For Fz, we also generated two alleles that truncate endogenous Fz upstream of the PBM, namely 573STOP (*fz^F1^*) and 568STOP (*fz^C11^*; Fig. 4A), but yet again, these proved to be viable and aphenotypic. However, immunostaining revealed a mild but statistically significant decrease in the proximodistal polarity of Fmi in *fz^F1^* mutant clones (Fig. S3). Intriguingly, we found that Fmi polarity is actually increased in double (*stan^F11^*; *fz^F1^*) and triple mutants (*dsh^C1^*; *stan^F11^*; *fz^F1^*) compared to the wt (Fig. S3). These polarity alterations of Fmi suggest that the C-terminal PBMs in these polarity proteins have a regulatory role in modulating proximodistal polarity; however, these PBMs are clearly not essential for polarity per se.

### Planar polarity relies on the DEP-dependent interaction between Dishevelled and Frizzled

In our CRISPR screen for C-terminal *fz* truncations, we also uncovered one (*fz^B4^*) that showed misoriented thoracic bristles and wing hairs (Fig. 4A) but neither leg nor axon guidance defects, reminiscent of *dsh^1^*, whose main defects are also in PCP (Fig. 1B). Furthermore, *fz^B4^* flies also exhibited reduced PS-Dsh (Fig. 4B), similar to *dsh^1^* and *fz^P21^* null mutants (Jones et al., 1996; Axelrod, 2001). Consistent with this, Dsh was no longer enriched at the apicolateral plasma membrane in pupal wings of *fz^B4^* mutants, even though the Fz expression levels were similar to the wt (Fig. 4C,D). This indicates an impaired ability of Fz^B4^ to recruit Dsh to the plasma membrane.

Recall that this recruitment step is mediated by direct binding of the Dishevelled DEP domain to the intracellular face of Frizzled (Gammons et al., 2016a; Pan et al., 2004; Tauriello et al., 2012). However, the deletion in *fz^B4^* truncates the C-terminal α-helix of Fz, which, by analogy to FZD4, is predicted to extend seven residues beyond the KTxxxW motif (Yang et al., 2018). Therefore, this truncation is likely to compromise the structural integrity of this helix, and thus of Fz^B4^ itself, which could impair its ability to recruit Dishevelled to the plasma membrane. We used a cell-based assay to confirm that SNAP–Fz^B4^ fails to recruit Dsh-DEP–GFP to the plasma membrane, in contrast to wt SNAP–Fz and SNAP–Fz^F1^, which do so efficiently (Fig. S4). Therefore, *fz^B4^* and *dsh^1^* both compromise the interaction between the two proteins, which could explain the polarity phenotypes caused by each of these lesions.

In summary, our study demonstrates that the Dishevelled PDZ domain is required exclusively for noncanonical Wnt signalling responses in multiple developmental contexts. However, the PCP phenotypes of *fz^B4^* and *dsh^1^* also highlight the importance of the DEP domain-dependent interaction between Frizzled and Dishevelled in planar cell polarity.

## MATERIALS AND METHODS

### Plasmids and antibodies

Dsh-DEP–GFP was generated by exchanging Dsh-DEP with DVL2-DEP from DEP–GFP (Gammons et al., 2016a). SNAP–Fz was generated by exchanging Fz with FZD5 from SNAP–FZD5 (Koo et al., 2012). Mutants were made from parental plasmids using standard site-directed mutagenesis procedures and were confirmed by sequencing.

The following antibodies were used for immunolabelling: 1:1000 rat anti-Dsh (Strutt et al., 2013), 1:300 affinity-purified rabbit anti-Fz (Bastock and Strutt, 2007), 1:1000 rat anti-Stbm (Strutt and Strutt, 2008), 3 µg/ml mouse anti-Fmi (Usui et al., 1999), 1:4000 anti-β-galactosidase (Cappel, 55976) and 1:1000 anti-SNAP (NEB, P9310). Secondary antibodies were 1:1000 Alexa Fluor 488-conjugated goat anti-mouse IgG (Invitrogen, A11029), 1:1000 Cy2-conjugated goat anti-rabbit IgG (Jackson ImmunoResearch, 111-225-144), 1:1000 Alexa Fluor 568-conjugated goat anti-rat IgG (Molecular Probes, A-11077), 1:1000 Alexa Fluor 568-conjugated goat anti-rabbit IgG (Molecular Probes, A-11036), 1:1000 Alexa Fluor 647-conjugated donkey anti-mouse IgG (Molecular Probes, A-31571) and 1:1000 Alexa Fluor 647-conjugated goat anti-rat IgG (Molecular Probes, A-21247). For western blotting, we used 1:200 affinity purified rabbit anti-Dsh (Strutt et al., 2006), 1:5000 mouse anti-β-actin AC-40 (Sigma, A4700) and 1:5000 anti-E-cadherin (Cell Signaling Technology, 24E10).

### CRISPR/Cas9 genome editing in flies

Standard strains of *Drosophila melanogaster* were used throughout. Our wt strain and the background of our mutants was w^1118^. The following mutant and transgenic strains were used (see also FlyBase): y w Ubx-FLP; arm-lacZ FRT80 and Ubi-GFP FRT19A; Ubx-FLP; w^1^, dsh^1^; Wnt4^C1^; Wnt4^EMS23^; dsh^v26^; omb.lacZ. Deletion and truncation mutants were generated essentially as described previously (van Tienen et al., 2017). The CRISPR design tool at https://crispr.mit.edu was used to design gRNAs targeting Dsh (PDZ: 5′-GGAGACGGTAATGATATTTA-3′ and 5′-GCTGGTGGTGGCCAAGTGCT-3′) or C-terminal PBMs (Dsh, 5′-CGGCAACAATACCAACGATC-3′; Fz, 5′-ACTAGACGTACGCCTGCGCC-3′; Fmi/Stan, 5′-GTATCCGTGATGCTTGTCAG-3′ and 5′-GGGCCCACTGACAAGCATCA-3′). Multiple alleles were obtained, and their lesions were determined by genomic sequencing.

### Fly strains and analysis

To observe retinal axons, *omb-lacZ* (Sato et al., 2006) was recombined with the desired mutant alleles. Paraformaldehyde-fixed late third-instar larval brains and eye imaginal discs were stained with anti-β-galactosidase, and confocal images were acquired with a Zeiss confocal microscope using a 40× objective. Fly legs, notums and wings were imaged with a Zeiss Axiophot microscope using a 10× objective. Pupal wings were dissected and fixed in 4% paraformaldehyde at 28 h after puparium formation (APF). Wings were blocked for 1 h in PBS containing 0.2% Triton X-100 (PTX) and 10% normal goat serum. Primary and secondary antibodies were incubated overnight at 4°C in PTX with 10% normal goat serum, and all washes were in PTX. After immunolabelling, wings were post-fixed in 4% paraformaldehyde in PBS for 30 min. Wings were mounted in 25 µl of PBS containing 10% glycerol and 2.5% DABCO, pH 7.5. Immunolabelled tissue was imaged on a Nikon A1R confocal microscope using a 60× objective. For western blots, 28 h APF pupal wings or late third-instar imaginal discs were dissected to generate whole cell lysates. Uncropped blots are shown in Fig. S5. Quantitation of planar polarity in pupal wings was performed as described previously (Strutt et al., 2016).

### Cell-based assays

HEK293T cells were obtained from ATCC (authenticated by STR DNA profiling) and regularly tested for mycoplasma infection. DEP recruitment assays in HEK293T cells were performed as previously described (Gammons et al., 2016a) and imaged on a Zeiss confocal microscope using a 40× objective.

### Protein purification and NMR spectroscopy

Proteins were purified essentially as described previously (Fiedler et al., 2011), NMR spectroscopy was performed as previously described (Gammons et al., 2016b).

## Supplementary Material

Supplementary information
