# Supplementary material for: Selective function of the PDZ domain of Dishevelled in noncanonical Wnt signalling
Source: J Cell Sci. 2022 May 31;135(11):jcs259547. doi: 10.1242/jcs.259547 (PMC9234668; doi:10.1242/jcs.259547)
Supplement: Supplementary information [file joces-135-259547-s1.pdf]

J. Cell Sci.: doi:10.1242/jcs.259547: Supplementary information

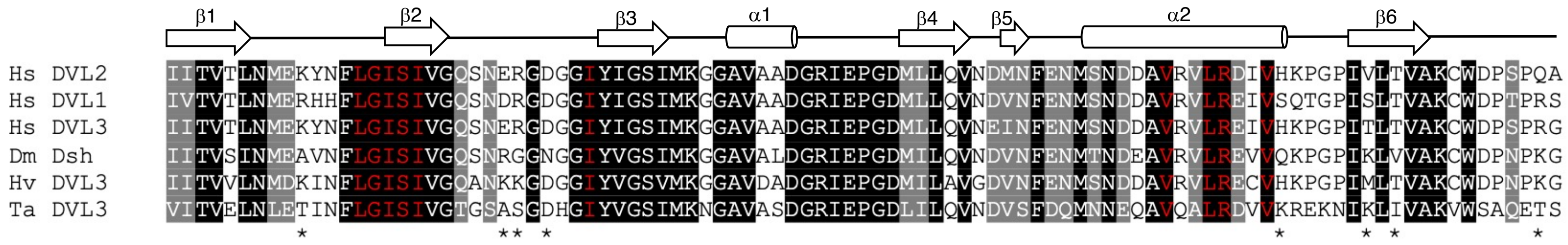

Fig. S1. (related to main **Fig. 3**)

Sequence conservation of the Dishevelled PDZ domain. Sequence alignments of the DVL PDZ domain across diverse animal species. Amino acids not similar between human (Hs) DVL2 and *Drosophila* (Dm) Dsh are indicated (*) although none are key peptide binding contacts (*red font*).

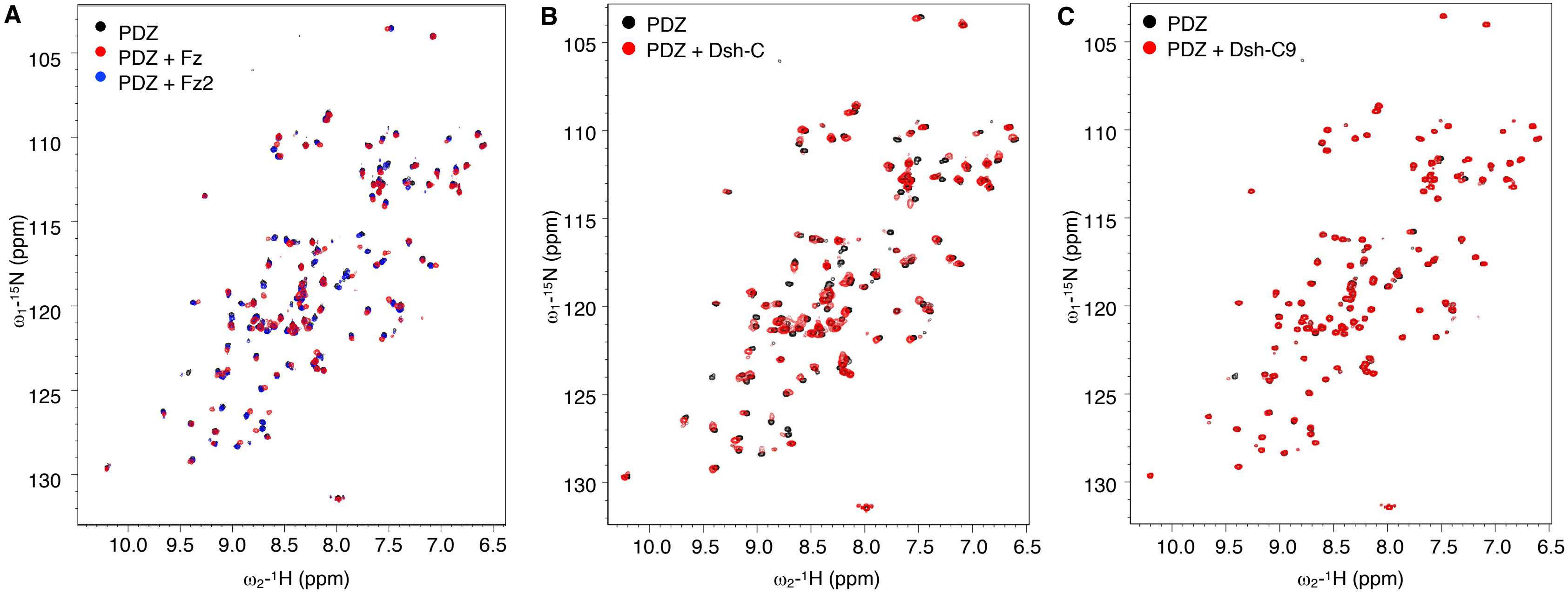

Fig. S2. (related to main **Fig. 3 and 4**)

Fz and Dsh-C but not Fz2 or Dsh-C9 peptide binding to PDZ. Overlay of HSQC spectra of 100μM ^{15}N-PDZ alone (*black*) or with 300μM Fz (*red*) or Fz2 (*blue*) (**A**) 150μM Dsh-C (*red*) (**B**) or with Dsh-C9 (*red*) (**C**).

Journal of Cell Science • Supplementary information

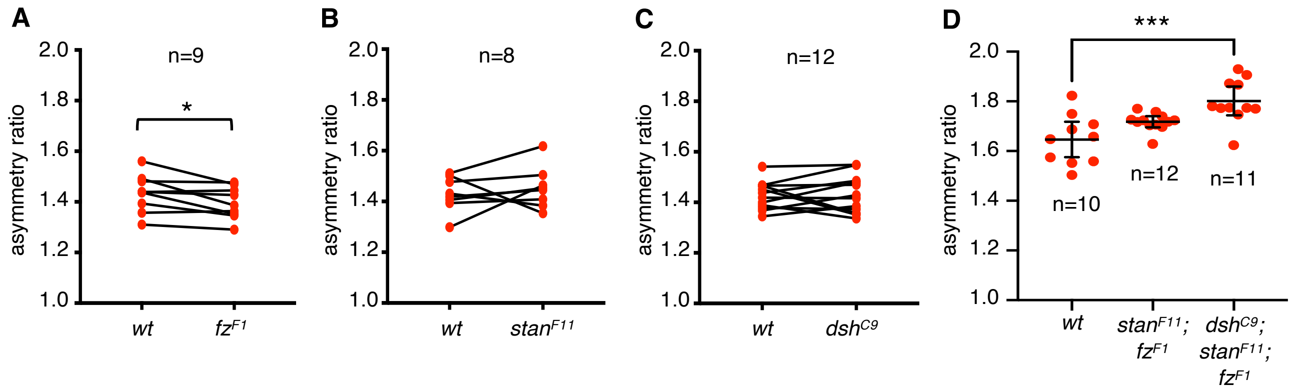

Fig. S3. (related to main **Fig. 4**)

PBM truncations modulate the polar distribution of Fmi. Quantitation of mean Fmi polarity in single, double and triple mutant wings compared to wt tissue, as indicated underneath graphs; paired T-tests were used to compare values in the same wing, *p<0.05 (**A-C**), one-way ANOVA with Dunnetts multiple comparisons test was used to compare mutant wings to wt wings immunolabelled in parallel, ***p<0.001; Error bars indicate 95% confidence intervals; N-numbers are indicated on graphs. The *fz*^{F1} truncation slightly reduces the polarised distribution of Fmi (**A**), whereas double and triple truncations increase polarity of Fmi compared to wt tissue (**D**), suggesting a modulatory and partially redundant role of these PBM in generating or maintaining proximodistal polarity. This could be somewhat indirect as the levels of Stbm and Fmi are also slightly reduced in these mutants.

Journal of Cell Science • Supplementary information

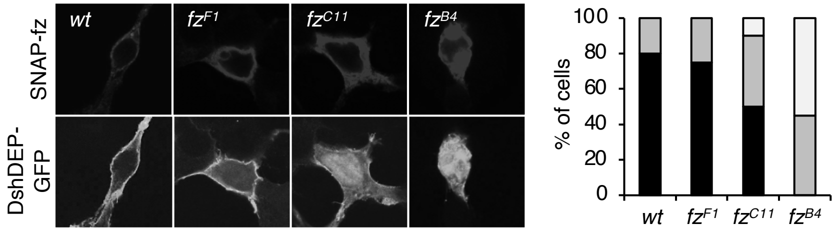

Fig. S4. (related to main **Fig. 4**)

DshDEP-GFP not recruited to SNAP-Fz^{B4}. Left, representative images of cells. Graph shows quantitation of wt or mutant Fz-dependent recruitment of Dsh DEP–GFP to the plasma membrane as strong (*black*), weak (*grey*); or none (*white*); 100 cells were scored in each case.

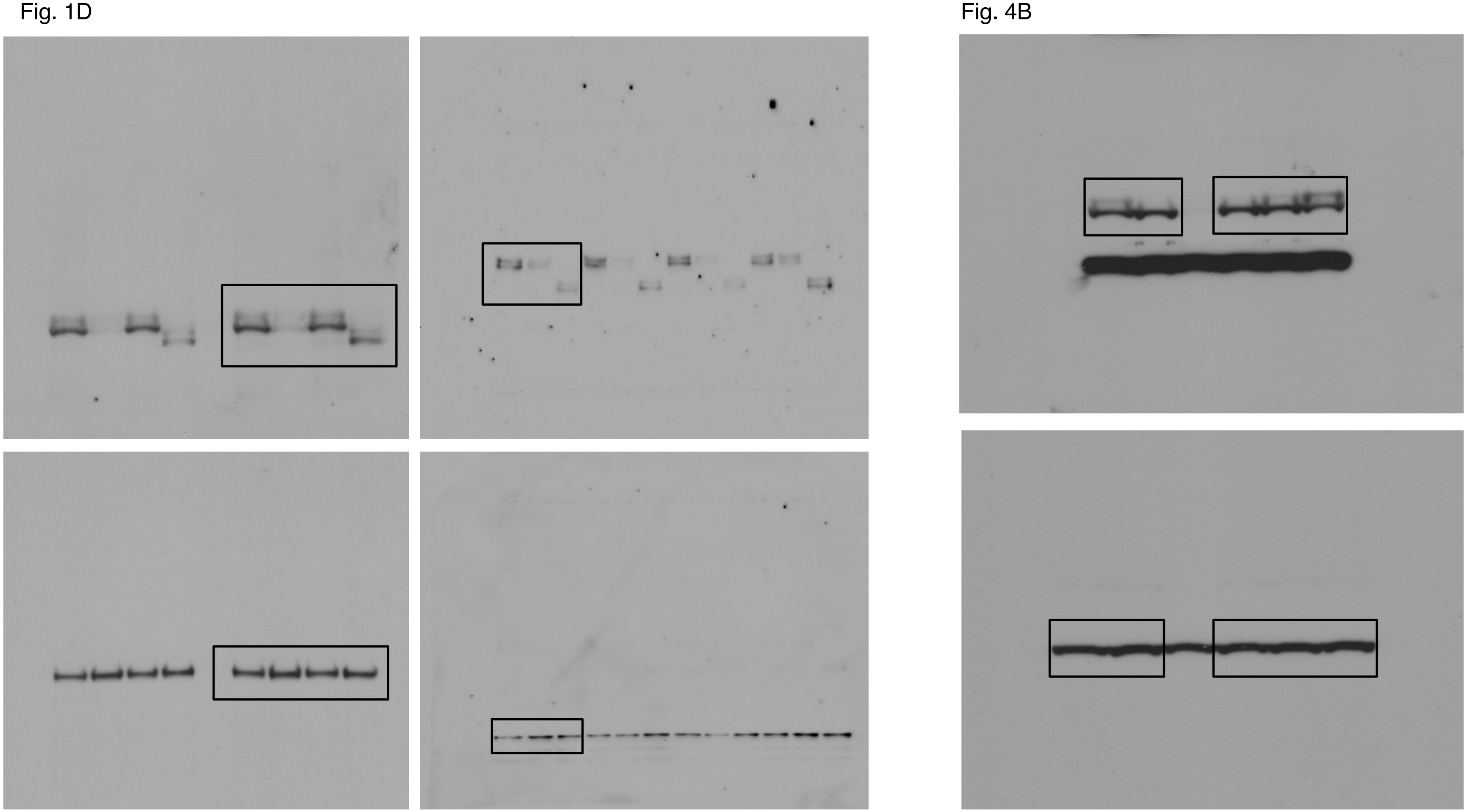

Fig. S5. Blot transparency (related to main **Fig. 1D and Fig. 4B**)

Journal of Cell Science • Supplementary information
